# Co-Culture with *Bifidobacterium catenulatum* Improves the Growth, Gut Colonization, and Butyrate Production of *Faecalibacterium prausnitzii*: In Vitro and In Vivo Studies

**DOI:** 10.3390/microorganisms8050788

**Published:** 2020-05-25

**Authors:** Heejung Kim, Yunju Jeong, Sini Kang, Hyun Ju You, Geun Eog Ji

**Affiliations:** 1 Department of Food and Nutrition, Research Institute of Ecology, Seoul National University, Seoul 08826, Korea; bonobono007@snu.ac.kr (H.K.); tanklov0@snu.ac.kr (Y.J.); kangsini@snu.ac.kr (S.K.); 2 Institute of Health and Environment, Graduate School of Public Health, Seoul National University, Seoul 08826, Korea; 3 Research Center, BIFIDO Co., Ltd., Hongcheon 25117, Korea

**Keywords:** *Faecalibacterium prausnitzii*, *Bifidobacterium catenulatum*, cross-feeding, anti-inflammation, butyrate

## Abstract

*Faecalibacterium prausnitzii* is a major commensal bacterium in the human gut. It produces short-chain fatty acids that promote intestinal health. However, the bacterium is extremely oxygen-sensitive, making it difficult to develop as a probiotic. To facilitate practical application of *F. prausnitzii*, we investigated factors that affect its growth and mammalian gut colonization. We evaluated cross-feeding interactions between *F. prausnitzii* and seven *Bifidobacterium* strains, and the anti-inflammatory properties of bacterial metabolites produced in co-culture, in vitro and in vivo. Co-culture of *F. prausnitzii* and *Bifidobacterium catenulatum*, with fructooligosaccharides as an energy source, resulted in the greatest viable cell-count and butyrate production increases. Further, the co-culture supernatant reduced the amount of proinflammatory cytokines produced by HT-29 cells and RAW 264.7 macrophages, an effect that was similar to that of butyrate. Furthermore, feeding mice both *Faecalibacterium* and *Bifidobacterium* enhanced *F. prausnitzii* gut colonization. Finally, feeding the co-culture supernatant decreased interleukin 8 levels in the colon and increased butyrate levels in the cecum in the dextran sodium sulfate-induced colitis mouse model. These observations indicate that the *Faecalibacterium*-*Bifidobacterium* co-culture exerts an anti-inflammatory effect by promoting *F. prausnitzii* survival and short-chain fatty acid production, with possible implications for the treatment of inflammatory bowel disease.

## 1. Introduction

Inflammatory bowel disease (IBD), including Crohn’s disease and ulcerative colitis, is characterized by inflammation and tissue damage in the distal ileum and colon. IBD causes chronic intestinal inflammation and is diagnosed in adolescents and children. The incidence of IBD in the wealthy western countries has been increasing since the mid-twentieth century. The incidence of IBD in Asia in the past 20 years has also increased, and IBD is currently recognized as an emerging global challenge [1,2,3,4].

The pathogenesis of IBD is driven by genetic predisposition and environmental factors, such as westernized diet and socioeconomic development. Gut dysbiosis is considered a novel pivotal factor associated with IBD pathogenesis and is characterized by a decreased diversity of the gut microbiota. Recent advances in next-generation sequencing allowed insight into the alteration of the gut microbiota, its composition, and physiological function in IBD [5]. Anti-inflammatory drugs used for treating IBD partially relieve the disease symptoms; however, the use of the drugs results in side effects such as headache and diarrhea, which lead to a poor quality of life [6]. This calls for novel treatment strategies, such as the use of probiotics.

*Faecalibacterium prausnitzii* is a member of *Clostridium* cluster IV within the Firmicutes phylum and is one of the most abundant commensal bacteria in the gut of healthy adults [7]. According to recent studies, *F. prausnitzii* exerts anti-inflammatory effect in vitro and in vivo. For instance, it strongly induces interleukin (IL)-10 production by the human peripheral blood mononuclear cells, and its culture supernatant reduces IL-8 secretion by HT-29 cells induced by tumor necrosis factor (TNF)-α [8,9]. In addition, administration of *F. prausnitzii* and its culture supernatant has a protective effect in a mouse model of dinitrobenzene sulfonic acid induced chronic colitis and dextran sodium sulfate (DSS)-induced colitis [10,11,12,13]. Importantly, the abundance of *F. prausnitzii* is reduced in individuals with IBD. Therefore *F. prausnitzii* serves as a potential biomarker for the diagnosis and prognosis of gut diseases [14,15].

*F. prausnitzii* produces butyrate by using butyrate kinase or butyryl CoA:acetate CoA transferase route, which exerts an anti-inflammatory effect by inhibiting the differentiation of T helper 17 cells [16,17]. Further, butyrate is a key energy source for the colonocyte and acts as an important regulator of gene expression, differentiation, and apoptosis of the host cell [18]. Butyrate and other short-chain fatty acids (SCFA) are also produced during fermentation of the dietary fiber in the colon. Diet—in particular, the type and quantity of substrates that survive the journey through the upper digestive system to the gut—affects the concentrations and relative proportions of SCFA in the gut [19,20].

Dietary fiber, such as resistant starch, inulin, and fructooligosaccharides (FOS), are major sources of energy for colonic bacteria [21]. The yield of bacterial metabolites from fermentable carbohydrates depends on the composition of the microbial community and the metabolic properties of the individual bacteria [22]. Cross-feeding bacteria use metabolites generated by fermentation by other bacteria as an energy source. For example, cross-feeding between *Bifidobacterium* and *Faecalibacterium* has been demonstrated in co-cultures in vitro. *Bifidobacterium* is the most abundant bacterial colonizer of the neonatal gut and is beneficial to the host intestinal health because of its metabolizing capacity to metabolize human milk oligosaccharides and exert immuno-modulatory functions in early age. Bifidobacteria produce lactate and acetate, but not butyrate [23]. In contrast, *F. prausnitzii* is an acetate-consuming and butyrate-producing bacterium and employs the butyryl CoA:acetate CoA transferase route for butyrate production [24]. It uses acetate produced by bifidobacteria in a starch- or FOS-containing medium, and this enhances its growth and butyrate production [25].

Despite the importance of *F. prausnitzii* as a next-generation probiotic candidate, to date, few studies using isolated strains have been conducted, with few functional approaches [7,11,13,26]. That is because this bacterium is difficult to cultivate even under anaerobic conditions. *F. prausnitzii* is an extremely oxygen-sensitive bacterium. It is particularly sensitive to exposure to air, surviving under such conditions for less than 2 min [25]. Therefore, intestinal delivery of *F. prausnitzii* remains challenging. Several approaches to increase the intestinal colonization and survival of *F. prausnitzii* have been attempted. Some methods for colonizing *F. prausnitzii* in the intestinal mucosa can be found in relation to other bacteria. For example, recent studies in adult gnotobiotic rodents revealed that *F. prausnitzii* colonizes the gut only with an a priori presence of *Bacteroides thetaiotaomicron* or *Escherichia coli* [26]. On the other hand, *F. prausnitzii* can grow in the presence of oxygen, provided that the growth medium contains flavins and cysteine or glutathione as an electron acceptor [27]. The interaction between bifidobacteria and bacteria from the *F. prausnitzii* has not yet been tested in vivo.

In the current study, we aimed to evaluate the effect of *F. prausnitzii* and bifidobacterial co-culture on the promotion of *F. prausnitzii* growth within the mammalian intestinal environment and butyrate production. We showed that acetate and degraded dietary fiber produced by different *Bifidobacterium* species promote the growth of *F. prausnitzii*, with cross-feeding also resulting in increased butyrate production. In line with the immunomodulatory properties of butyrate, the co-culture supernatant of *F. prausnitzii* and *Bifidobacterium catenulatum* exerted anti-inflammatory effects in macrophages and a DSS-induced colitis mouse model. In addition, administration of *B. catenulatum* or the co-culture supernatant to mouse enhanced gut colonization of *F. prausnitzii* in vivo as assessed by 16S rRNA sequencing and real-time quantitative PCR. These findings indicate that the co-culture of *B. catenulatum* and *F. prausnitzii* in fortified FOS media can be a useful strategy to enhance the growth and colonization of *F. prausnitzii*, increase butyrate production, and alleviate the symptoms of DSS-induced colitis.

## 2. Materials and Methods

### 2.1. Bacterial Strains and Culture

The fecal isolates *F. prausnitzii* ATCC 27768 and A2-165 and seven *Bifidobacterium* strains were used. Commercial strains of *F. prausnitzii* ATCC 27768, *F. prausnitzii* A2-165, and *B. catenulatum* KCTC 3221 were purchased from American Type Culture Collection (ATCC, Manassas, VA, USA), DSMZ-German Collection of Microorganisms and Cell Cultures (DSMZ, Braunschweig, Germany), and Korean Collection for Type Cultures (KCTC, Daejeon, Korea), respectively. Six *Bifidobacterium* strains were isolated from fecal samples of healthy infants in Korea and identified by 16rRNA gene sequencing. *F. prausnitzii* ATCC 27768 and *F. prausnitzii* A2-165 were cultured anaerobically for 24 h at 37 °C in modified reinforced clostridial broth in Hungate tubes. *B. catenulatum* KCTC 3221, *B. bifidum* BGN4*, B. logum* RD72, *B. animalis* ATCC 25557, *B. animalis* subsp. *lactis* RD68*, B. animalis* subsp. *lactis* SH5, and *B. animalis* subsp. *lactis* ad011 were incubated anaerobically in de Man, Rogosa, and Sharpe (MRS) medium (Difco, Detroit, MI, USA) supplemented with 0.05% (*w*/*v*) l-cysteine for 18 h at 37 °C.

### 2.2. Assessment of Carbohydrate Utilization and Bacterial Growth

Co-culture experiments with selected strains, and in the presence of specific carbon sources, were performed to study the interaction between *F. prausnitzii* ATCC 27768 and *Bifidobacterium* species. For each strain, overnight culture was inoculated to 1% (*v*/*v*) in 10 mL Yeast extract, Casitone, FOS (YCFOS) medium [25]. The co-cultures were set up by mixing the different bacteria in a 1:1 ratio. Yeast extract, casitone, and fatty acid (YCFA) broth without SCFA was supplemented with glucose, starch, inulin, or FOS (3.5 g/L for each carbohydrate). All manipulations were performed in an anaerobic chamber (Coy Lab, Grass Lake, MI, USA) at 37 °C under an atmosphere of 80% N_2_, 10% H_2_, and 10% CO_2_. Liquid medium in Hungate tube was placed in an anaerobic chamber immediately after autoclaving. LYBHI (brain-heart infusion medium supplemented with 0.5% yeast extract) (Difco, Detroit, MI, USA) agar plates were prepared and equilibrated in an anaerobic chamber for 24 h before use. Bacterial growth in monoculture and co-culture was monitored by measuring culture optical density (OD_650 nm_), viable cell counts, pH, and SCFA production (Section 2.2.1 and Section 2.2.2). For Opitical density (OD) measurements and SCFA analysis, the cultures were sampled after 0, 9, and 24 h of incubation. The bacterial supernatants used in in vitro cell culture experiments were collected after cultivation for 24 hr of *Faecalibacterium* and *Bifidobacterium*, respectively. The samples were centrifuged, and the medium was immediately stored at −80 °C until use.

#### 2.2.1. pH Measurements and Viable Cell Counts

All procedures were performed in an anaerobic chamber. Monocultures and *F. prausnitzii* ATCC 27768 co-cultures with *B. catenulatum* KCTC 3221 or *B. animalis* ATCC 25527 were incubated in YCFOS medium without SCFA or plated on LYBHI medium at 37 °C [28]. The initial medium pH was determined by using a pH meter (model K2000-pH; Istek, Inc., Seoul, Korea). The pH was measured after 0, 7, 18, and 24 h of incubation. For viable cell counts, cells from the monocultures and co-cultures were plated on LYBHI agar medium and incubated at 37 °C. The resultant colonies were counted after 2 d. *F. prausnitzii* forms transparent colonies, whereas bifidobacteria form yellow colonies. The data are expressed as log10[colony-forming units (CFU)]/mL.

#### 2.2.2. SCFA Determinations

SCFA levels were determined as previously described [29]. Briefly, organic acids, such as acetate, lactate, formate, and butyrate, were analyzed by high-performance liquid chromatography. A YL9100 HPLC system (Young-lin, Anyang, Korea), equipped with an RI detector and Aminex HPX-87H column (300 × 7.8 mm; Bio-Rad Laboratories, Hercules, CA, USA), was used. Cecum samples (100 mg) were homogenized in phosphate-buffered saline (400 μL). Then, 25% (w/v) meta-phosphoric acid (Sigma-Aldrich, St. Louis, MO, USA) was added to the homogenate at a 1:5 ratio, and the samples were incubated at room temperature for 30 min. The samples were centrifuged at 15,000× *g* for 15 min, and the supernatants were stored at −80 °C until further processing [30]. The liquid culture medium was centrifuged at 18,000× *g* for 15 min, and the supernatant was then filtered through a 0.2 μm filter. For the analysis, 0.005 M sulfuric acid (J.T. Baker, Phillipsburg, MA, USA) was the mobile phase, with a flow rate of 0.6 mL/min. The concentration of each organic acid was normalized to the corresponding concentrations of external SCFA standards and is expressed in mM.

### 2.3. Cell Culture and Determination of Immunomodulatory Properties of Culture Supernatants

HT-29 cells and RAW 264.7 macrophages were obtained from Korea Cell Line Bank (KCLB, Daejeon, Korea). HT-29 cells (1 × 10^5^ cells/well) and RAW 264.7 macrophages (1 × 10^5^ cells/well) were seeded into 24-well plates in Dulbecco’s modified Eagle’s medium supplemented with 10% heat-inactivated fetal bovine serum (Welgene Inc., Daegu, Korea) and 1% antibiotic antimycotic solution (Sigma-Aldrich, St. Louis, MO, USA) and cultured for 3–4 d at 37 °C under 5% CO_2_. Then, the cells were stimulated with 0.1 μg/mL lipopolysaccharide (LPS) (Sigma-Aldrich St. Louis, MO, USA). Next, 10% (*v*/*v*) of bacterial supernatant (pH 7.0) was added in Dulbecco’s modified Eagle’s medium in a total volume of 1 mL. The plates were incubated for 6 h at 37 °C under 5% CO_2_. All samples were analyzed in triplicate. After the incubation, cell culture supernatants were collected and stored at −80 °C until further analysis. IL-6, TNF-α, and IL-8 levels were determined by using enzyme-linked immunosorbent assay kits (BD Biosciences, San Jose, CA, USA) according to the manufacturer’s instructions [8].

### 2.4. Animals and Experimental Design

The experiments were approved by the ethics committee of Seoul National University (Institutional Animal Care and Use Committee, experiment 1 approval number, approval date: SNU-181127-11-1, 2019-03-25; experiment 2 approval number, approval date: SNU-191120-3, 2019-11-20). The mice were maintained at the animal facility of Seoul National University. Two different experiments were performed. In experiment 1, 24 male C57BL/6 mice (5–6-week-old, 18–21 g; OrientBio, Sungnam, Korea) were used. Mice were acclimatized for 1 week prior to the experiment. The animals were separated into four groups (*n* = 6 per group, *n* = 2 per cage): mice administered with live *F. prausnitzii* (F group), mice administered with live *F. prausnitzii* and *B. catenulatum* (fed both bacteria (FB) group), mice administered with live *B. catenulatum* (B group), and mice administered with sterile saline (C group). Live bacteria were administered in 200 μL doses (see Section 2.4.1), daily, by gavage, for 14 d. All mouse feces were collected before sacrifice (*n* = 6, respectively), and fecal bacterial abundance was determined, as described in Section 2.4.3 and Section 2.4.4. In experiment 2, 45 female C57BL/6 mice (8-week-old, 18–21 g, OrientBio, Sungnam, Korea) were used. The animals were separated into five groups (*n* = 7–9 per group, *n* = 2–3 per cage): untreated mice (*n* group), mice that were administered DSS and YCFOS medium (M group), mice that were administered DSS and supernatant from *F. prausnitzii* and *B. catenulatum* co-culture (FB group), mice that were administered DSS and supernatant from *F. prausnitzii* culture (F group), and mice that were administered DSS and supernatant from *B. catenulatum* culture (B group). The groups with DSS-induced colitis received 2% (*w*/*v*) DSS (molecular mass, 36,000–50,000 kDa; MP Biomedicals, Santa Ana, CA, USA) in drinking water, ad libitum, for 7 d, followed by 2 d of plain tap water. The mice were given bacterial supernatant by gavage (see Section 2.4.1), and bacterial supernatants were administered in 200 μL doses and were weighed daily. The animals were sacrificed on day 9, and organs were harvested for analysis. Six samples per group were processed for histopathology and reverse-transcription quantitative polymerase chain reaction (RT-qPCR) (Section 2.4.2).

#### 2.4.1. Sample Preparation for Administration

For experiment 1, *F. prausnitzii* ATCC 27768 was cultured in a modified reinforced clostridial broth for 24 h at 37 °C. *B. catenulatum* KCTC 3221 was grown in MRS broth with L-cysteine for 18 h at 37 °C. The bacteria were harvested by centrifugation at 8000× *g* for 10 min at 4 °C, washed in phosphate-buffered saline, and re-suspended in sterile saline. The cells were administered orally (1 × 10^9^ CFU) in 200 μL sterile saline. *F. prausnitzii* (1 × 10^9^/100 μL) and *B. catenulatum* (1 × 10^9^/100 μL) were administered together to the FB group. For experiment 2, *F. prausnitzii* A2-165 and *B. catenulatum* KCTC 3221 were grown in YCFOS medium at 37 °C for 24 h. Supernatant was collected from 10^7^–10^8^ CFU/mL culture by centrifugation. The supernatant was lyophilized and stored at −80 °C. The lyophilizate was reconstituted in a 20-times concentrated volume.

#### 2.4.2. DNA Extraction and Real-Time qPCR Amplification

Total genomic DNA from the stool and cecum were extracted and purified by using a QIAamp Fast DNA Stool Mini kit (Qiagen, Germantown, MD, USA) according to the manufacturer’s instructions, followed by bead beating using a TissueLyser system (Qiagen, Germantown, MD, USA). Bacterial genomic DNA was quantified using a Qubit 3.0 fluorometer (Thermo Fisher Scientific, Waltham, MA, USA) in conjunction with the Qubit dsDNA High Sensitivity (HS) Assay Kit. DNA purified from each sample was used for RT-qPCR and 16S rRNA gene sequencing (Section 2.4.4). The abundance of *Faecalibacterium* and *Bifidobacterium* among the fecal microbiota was quantified by RT-qPCR, using the SYBR^®^ Premix Ex Taq™ and StepOne™ Real-Time PCR System. *Faecalibacterium* and *Bifidobacterium* abundances were quantified using the following primers: Frau223F, 5′-GAT GGC CTC GCG TCC GAT TAG-3′, and Frau420R, 5′-CCG AAG ACC TTC TTC CTC C-3′ [31]; Bif164F, 5′-GGG TGG TAA TGC CGG ATG-3′, and Bif601R, 5′-TAA GCC ATG GAC TTT CAC ACC-3′ [32], respectively. The PCR reaction was performed as follows: 95 °C for 30 s, followed by 40 cycles at 95 °C for 30 s, 95 °C for 5 s, and 60 °C for 34 s. The relative abundance of *Faecalibacterium* and *Bifidobacterium* was calculated using the cycle threshold method.

#### 2.4.3. 16S rRNA Gene Amplification and Sequence Analysis

Bacterial genomic DNA isolated from feces was analyzed by next-generation sequencing as previously described [29]. 16S rRNA gene amplification and index PCR were performed following the Illumina 16S Metagenomic Sequencing Library preparation guide (Illumina, San Diego, CA, USA). The V3–V4 region of the bacterial 16S rRNA gene was PCR-amplified using the primers 16S Amplicon PCR Forward primer, 5′-TCG TCG GCA GCG TCA GAT GTG TAT AAG AGA CAG CCT ACG GGN GGC WGC AG-3′, and 16S Amplicon PCR Reverse primer, 5′-GTC TCG TGG GCT CGG AGA TGT GTA TAA GAG ACA GGA CTA CHV GGG TAT CTA ATC C-3′. The amplicons were attached to specific barcode sequences to compile the pooled library using a Nextera XT index kit (Illumina San Diego, CA, USA). The PCR products were purified using AMPure XP beads (Beckman Coulter, Pasadena, CA, USA). Diluted DNA (4 nM) was pooled into a library. The pooled library was denatured with 1N NaOH and diluted to 9 pM in HT1 hybridization buffer (Illumina San Diego, CA, USA). A PhiX control library (Illumina San Diego, CA) was also denatured and diluted. The pooled library was mixed with the PhiX control (30%, *v*/*v*) and loaded onto a MiSeq^®^ version 2 (500 cycle) reagent cartridge (Illumina San Diego, CA, USA) for sequencing. Paired-end FASTQ files were quality-filtered, and data were processed using Quantitative Insights Into Microbial Ecology 2 (QIIME2) (version 2019.7, https://qiime2.org). Alpha- and beta-diversity analysis and operational taxonomic unit assignment were done using the q2-diversity plugin within QIIME2. Linear discriminant analysis (LDA) effect size analysis (LEfSe) was then performed.

#### 2.4.4. Histological Analysis and RT-qPCR of the Colon Tissue

For RT-qPCR, total RNA was isolated from a 30 mg sample of colon (stabilized in RNAlater) using an RNeasy mini kit (Qiagen, Germantown, MD, USA). RNA concentration was determined by NanoDrop. The first-strand complementary DNA was synthesized using a reverse-transcription kit (Invitrogen, Carlsbad, CA, USA). The complementary DNA was amplified using SYBR^®^ Premix Ex Taq™ (Takara, Dalian, China). PCR was performed using the StepOne™ Real-Time PCR System (Applied Biosystems, Foster City, CA). The following qPCR primers were used: for *Il8*, IL-8 Forward, 5′-CTA GGC ATC TTC GTC CGT CC-3′, and IL-8 Reverse, 5′-TTC ACC CAT GGA GCA TCA GG-3′ [33]; for *Il6*, IL-6 Forward, 5′-CTG CAA GAG ACT TCC ATC CAG-3′, and IL-6 Reverse, 5′-AGT GGT ATA GAC AGG TCT GTT GG-3′ [34]; for *Tnfa*, TNF-a Forward, 5′-CAT CTT CTC AAA ATT CGA GTG ACA A-3′, and TNF-a Reverse, 5′-TGG GAG TAG ACA AGG TAC AAC CC-3′ [35]; and for *Gapdh*, GAPDH Forward, 5′-AGG TCG GTG AAC GGA TTT G-3′, and GAPDH Reverse, 5′-TGT AGA CCA TGT AGT TGA GGT CA-3′ [36], respectively. Relative mRNA levels were determined using the comparative cycle threshold method. *Gapdh* was used as the reference gene.

For the histological analysis, the colon was fixed in 10% buffered formalin. The tissue was then dehydrated and embedded in paraffin. Then, 5 mm sections were prepared and stained with hematoxylin and eosin (H&E). The severity of colitis in each section was semi-quantified using a scoring system of 0 to 4: 0, normal crypt architecture; 1, crypt shortening, loss of the basal one-third portion of the crypt, with the lamina propria prominent and minimal inflammatory changes; 2, crypt shortening, loss of the basal two-thirds portion of the crypt and thinning of the epithelium with mild inflammatory changes; 3, loss of the entire crypt, with an intact epithelial layer and moderate inflammatory changes; and 4, loss of the entire crypt and epithelial layer (erosion), with severe inflammatory changes.

### 2.5. Statistical Analyses

All statistical analyses were performed using GraphPad Prism version 8.0 (GraphPad, La Jolla, CA, USA). All graphics were prepared using the same program. The Kruskal-Wallis test and a non-parametric one-way analysis of variance were used for differential relative abundance analysis. Parametric analysis of variance was used for statistical analysis of several groups. All data are presented as the mean ± SEM; *p* < 0.05 was considered to indicate statistical significance.

## 3. Results

### 3.1. Effects of Mono- and Co-Culture, and Different Carbon Sources on Bacterial Growth, SCFA Production, Culture pH, and Viable Cell Counts

#### 3.1.1. Growth of Bacteria in Mono- and Co-Cultures in the Presence of Different Carbon Sources

**Figure 1:**
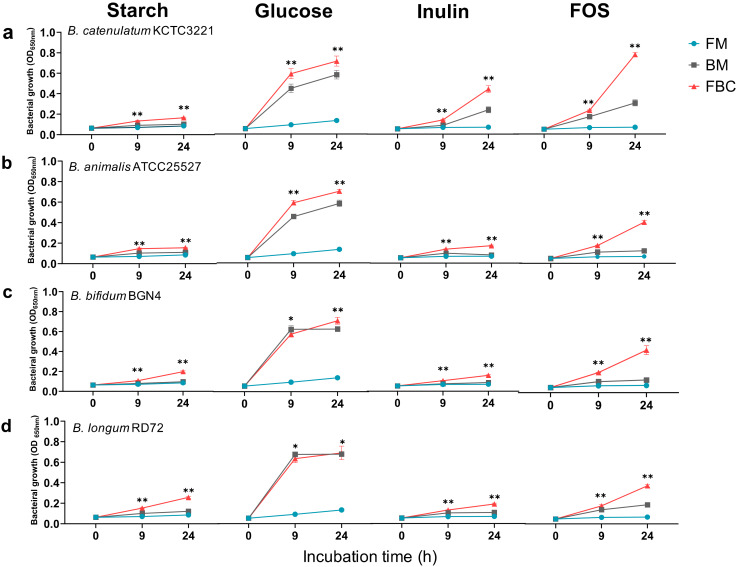
Growth of *F. prausnitzii* ATCC 27768 with different *Bifidobacterium* species in liquid culture in the presence of various carbohydrates. Culture OD_650_ was determined at various time points after inoculation. *F. prausnitzii* growth in monoculture and co-culture with *B. catenulatum* KCTC 3221 (**a**), *B. animalis* ATCC 25527 (**b**), *B. bifidum* BGN4 (**c**), and *B. longum* RD72 (**d**) is shown. All growth experiments were performed in the YCFA medium without SCFA supplemented with starch, glucose, inulin, or FOS, for 24 h. The data are presented as the mean ± standard deviation (*n* = 4). Statistically significant differences vs. FM group are denoted (* *p* < 0.05, ** *p* < 0.01). FM, monoculture of *F. prausnitzii*; BM, monoculture of *Bifidobacterium*; FBC, co-culture of *F. prausnitzii* and *Bifidobacterium*.

In the current study, we aimed to identify the factors that affect *F. prausnitzii* growth in order to enable development of this bacterium as a probiotic. We first investigated the growth (OD_650_) of *F. prausnitzii* ATCC 27768 and different Bifidobacteria in monoculture and co-culture, in the presence of different carbon sources (Figure 1). *F. prausnitzii* did not grow well on the carbon sources tested in monoculture. By contrast, *Bifidobacterium* growth in monoculture was carbon-source-dependent. Further, the stimulation of general bacterial growth in co-culture was also carbon-source-dependent. All bacteria grew best on glucose, with second-best growth on FOS. This indicated that the bacteria grew better in the presence of a short-chain sugar substrate (FOS) or monosaccharides (glucose) than in the presence of long-chain sugars, such as starch and inulin. After 24 h co-culture, the relative growth on FOS was 54.1–109% that of growth on glucose (23.1–62.3% for inulin and 22–29.9% for starch). Compared with the growth on glucose, *F. prausnitzii* and *B. catenulatum* co-culture showed good growth on inulin (62.3%) and FOS (109%). In addition, *B. catenulatum* grew better on inulin and FOS than the other *Bifidobacterium* species tested. Co-culture on FOS resulted in better growth than co-culture on inulin, with the best growth for *F. prausnitzii* co-culture with *B. catenulatum* (OD_650_ = 0.79 ± 0.02). For *B. catenulatum* KCTC 3221 and *B. animalis* ATCC 25527, the overall growth of the co-culture was better than that of *Bifidobacterium* and *F. prausnitzii* alone. However, in the glucose-containing medium, *B. bifidum* BGN4 and *B. longum* RD72 did not show noticeable changes in growth between monoculture and co-culture. We also observed some strain-specific differences in the growth of *B. animalis* species (Figure S1). Collectively, these observations highlight the strain- and species-dependent differences in the growth of bifidobacteria.

#### 3.1.2. SCFA Production by Bacteria in Mono- and Co-Culture in the Presence of Different Carbon Sources

**Figure 2:**
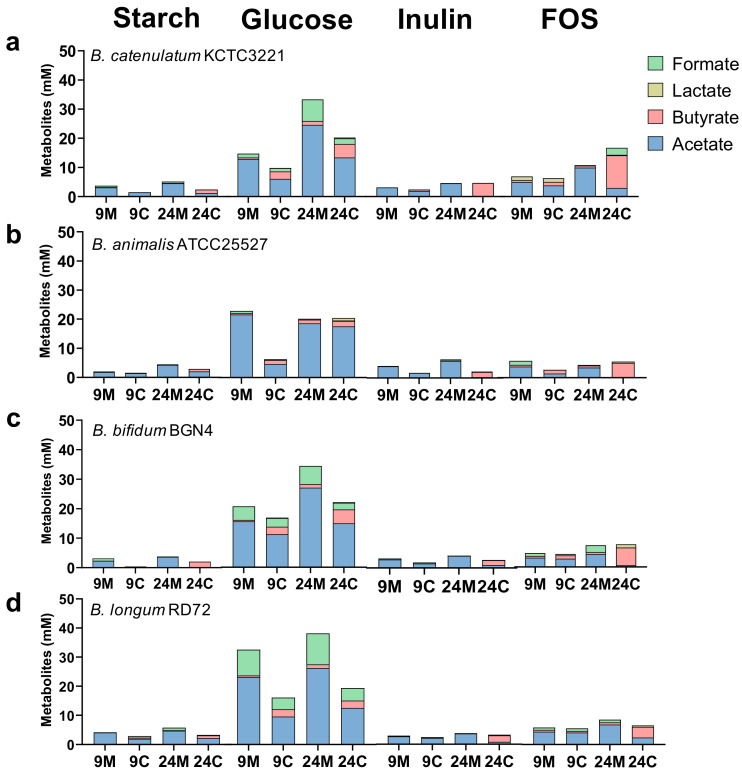
Production of SCFA by bacteria in monoculture and co-culture. Concentrations of SCFA (acetate, lactate, formate, and butyrate) were determined 9 and 24 h after inoculation. Data for *F. prausnitzii* in monoculture and in co-culture with *B. catenulatum* KCTC 3221 (**a**), *B. animalis* ATCC 25527 (**b**), *B. bifidum* BGN4 (**c**), and *B. longum* RD72 (**d**) are shown. All experiments were performed in YCFA medium without SCFA supplemented with starch, glucose, inulin, or FOS. The data are presented as the mean (*n* = 3). 9M, metabolites from *F. prausnitzii* and *Bifidobacterium* monocultures after 9 h*;* 9C, metabolites in *F. prausnitzii* and *Bifidobacterium* co-culture after 9 h; 24M, metabolites from *F. prausnitzii* and *Bifidobacterium* monocultures after 24 h; 24C, metabolites in *F. prausnitzii* and *Bifidobacterium* co-culture after 24 h.

We next determined the SCFA levels in different mono- and co-cultures after 9 and 24 h of cultivation (Figure 2). Only a small amount of SCFA was produced in the starch-containing medium, reflecting the weak growth of bacteria in this medium. Most SCFA were produced in the glucose-containing medium, which also reflected the highest bacterial growth in this medium. The main SCFA produced on glucose was acetate. Further, more acetate was present in co-culture after 24 h than after 9 h, regardless of the *Bifidobacterium* species used. *B. catenulatum* KCTC 3221, *B. bifidum* BGN4, and *B. longum* RD72 produced lactate. The bacteria produced less lactate in co-culture than in monoculture. *B. catenulatum* KCTC 3221 and *B. bifidum* BGN4 produced more butyrate than *B. animalis* ATCC 27768 and *B. longum* RD72 (4.61 ± 1.50 mM and 4.85 ± 0.37 mM vs. 1.85 ± 0.69 mM and 2.44 ± 0.96 mM, respectively). In the inulin-containing medium, the bacteria produced most acetate in monoculture, while butyrate was the major SCFA after 24 h of co-culture. We noted similar trends in the FOS-containing medium, although this medium generally supported higher SCFA production than the inulin-containing medium. The main SCFA produced on FOS was butyrate after 24 h of co-culture with *F. prausnitzii* (11.17 ± 0.05 mM, 4.99 ± 0.71 mM, 3.69 ± 0.56 mM, and 6.19 ± 1.26 mM, respectively). Further, butyrate concentrations were significantly higher in every Bifidobacteria co-cultures than in the corresponding *F. prausnitzii* monocultures. In addition, unlike on glucose, acetate levels after 24 h of co-culture were lower than those after 9 h of co-culture. We observed the greatest increase in butyrate levels between 9 and 24 h incubation in *F. prausnitzii* ATCC 27768 co-culture with *B. catenulatum* KCTC 3221 in the presence of FOS (1.24 ± 1.40 mM vs. 11.17 ± 0.05 mM, respectively). Further, different *B. animalis* strains produced different amounts of SCFA (Figure S2). *B. animalis* subsp. *lactis* RD68 and *B. animalis* subsp. *lactis* SH5 produced more butyrate in the glucose-containing medium than in the FOS-containing medium after 24 h of co-culture with *F. prausnitzii* (6.56 mM, 8.66 mM, 6.12 mM, and 5.51 mM in respective *B. animalis* strains). Finally, *B. animalis* subsp. *lactis* Ad011 produced large amounts of lactate on all carbon sources tested (Figure S2). Taken together, these observations indicate that the effect of co-culture on SCFA production was species-/strain-specific and dependent on the type of carbon sources.

#### 3.1.3. Effect of *F. prausnitzii* ATCC 27768 Co-Culture with *B. catenulatum* KCTC 3221 or *B. animalis* ATCC 25527 in a Medium Containing FOS on Culture pH and Viable Cell Counts

**Figure 3:**
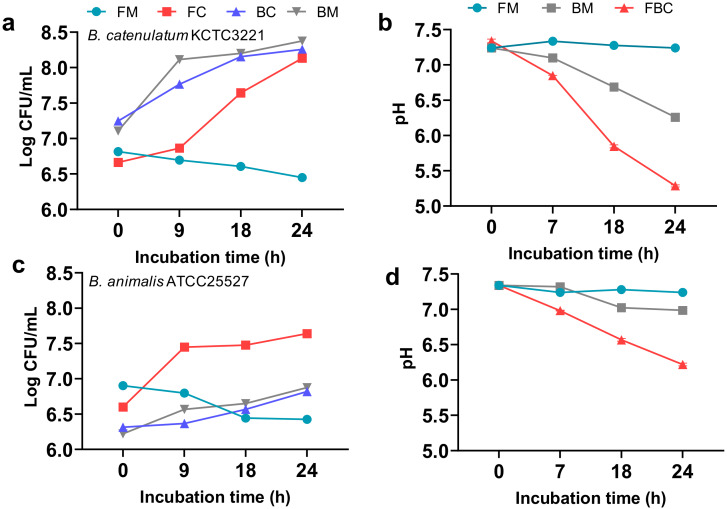
Changes in the viable cell number and pH of cultures of *F. prausnitzii* and *B. catenulatum* KCTC 3221 or *B. animalis* ATCC 25527 in YCFOS medium. Data for *F. prausnitzii* in monoculture or co-culture with *B. catenulatum* KCTC 3221 (**a,b**) or *B. animalis* ATCC 25527 (**c,d**) in YC medium supplemented with FOS are shown. Samples were analyzed at the indicated time intervals. Cell numbers were determined by viable counts, as CFU/mL, after plating on LYBHI agar (**a**,**c**). The pH changes are plotted in (**b**,**d**). The data are presented as the mean ± standard deviation (*n* = 3). FM, *F. prausnitzii* in monoculture; BM, *Bifidobacterium* in monoculture; FC, *F. prausnitzii* in co-culture; BC, *Bifidobacterium* in co-culture;FBC, co-culture of *F. prausnitzii* and *Bifidobacterium.*

*B. catenulatum* KCTC 3221 and *B. animalis* ATCC 25527 showed distinct growth patterns in the FOS-containing media. We followed up these observations by viable cell-count and pH analyses in the FOS medium (Figure 3). The number of bacteria in *F. prausnitzii* monoculture decreased after 24 h (6.82 to 6.45 CFU/mL) (Figure 3a,c). By contrast, in co-culture with *Bifidobacterium*, the number of *F. prausnitzii* cells increased over time. In the case of B. *catenulatum* KCTC 3221, both the *Bifidobacterium* and *F. prausnitzii* grew well in co-culture. Further, *F. prausnitzii* in co-culture grew more rapidly between 18 and 24 h than during the initial 9 h (logCFU/mL change of 7.64 to 8.13 vs. 6.66 to 6.87, respectively). Interestingly, although the culture pH dropped substantially in the *F. prausnitzii* co-culture with *B. catenulatum* KCTC 3221 (pH 7.34 to 5.28) (Figure 3b), *F. prausnitzii* grew well under these conditions. In co-culture with *B. animalis* ATCC 25527, although the *Bifidobacterium* did not grow well, it stimulated the growth of *F. prausnitzii*. *B. animalis* subsp. *lactis* RD68 showed a similar growth-promoting effect on *F. prausnitzii* (Figure S3). The culture pH in *F. prausnitzii* co-culture with *B. animalis* subsp. *lactis* RD68 did not decrease below 6.0, and this represented the low growth of RD68 strain in the FOS-containing medium. These data suggested that the increased viable cell number of *F. prausnitzii* in the *F. prausnitzii* and *B. catenulatum* co-culture in the FOS-containing medium was related to the highest amount of butyrate produced by *F. prausnitzii*, and *B. catenulatum* facilitated it with rapid digestion of dietary fiber and acetate production.

### 3.2. Comparison of the Fecal Microbiota of Normal Mice Fed F. prausnitzii ATCC 27768 and B. catenulatum KCTC 3221

**Figure 4:**
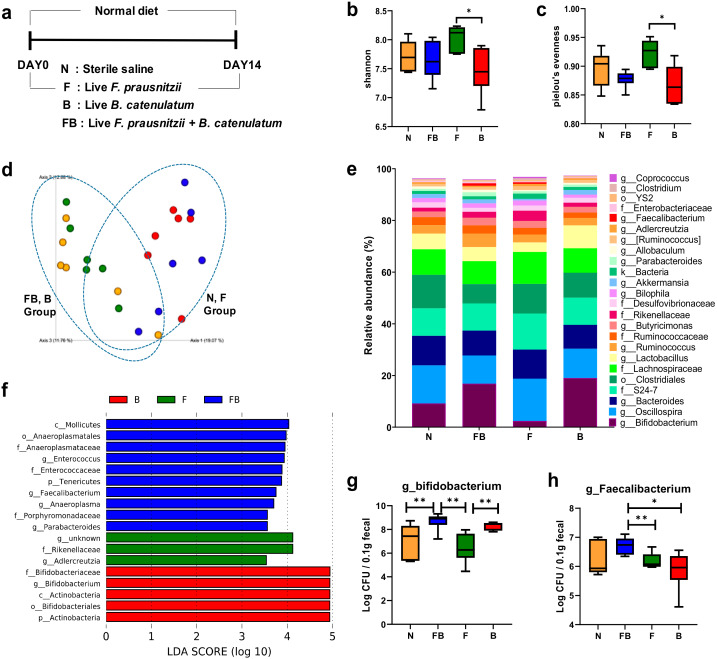
Fecal microbial composition in normal C57B6 mice fed *F. prausnitzii* and *B. catenulatum*. The microbiome was analyzed in mouse stool samples (6 mice per group). The mice were administered live bacteria or saline for 14 d (**a**). The alpha-diversity indices of microbial community richness and evenness are shown in (**b**) and (**c**). The beta-diversity based on the Bray-Curtis distance is shown in (**d**). The average relative abundance at the genus level (taxa with relative abundance >0.5%) is shown in (**e**). Significantly different taxa in the B (red), F (green), and FB (blue) groups, determined by LEfSe analysis (threshold >2.0), are shown in (**f**). The abundance of *F. prausnitzii* (**g**) and *Bifidobacterium* (**h**) in the feces was detected by RT-qPCR. Statistically significant differences are shown (* *p* < 0.05, ** *p* < 0.01). N, normal mouse; FB, mice administered *F. prausnitzii* (1 × 10^9^ CFU/mL) and *B. catenulatum* (1 × 10^9^ CFU/mL); F, mice administered *F. prausnitzii* (1 × 10^9^ CFU/mL); B, mice administered *B. catenulatum* (1 × 10^9^ CFU/mL).

We next investigated whether *B. catenulatum* increased the survival of *F. prausnitzii* in a normal mouse gut. We fed the mice bacteria for 2 weeks and then extracted bacterial genomic DNA from the feces and performed 16S rRNA amplicon sequencing and RT-qPCR analysis (Figure 4a). As shown in Figure 4, the intake of bacteria affected the composition of total fecal microbiota in mouse. Further, the number of *Faecalibacterium* in the feces of animals fed *F. prausnitzii* and *B. catenulatum* was significantly higher than that in the feces of animals fed *F. prausnitzii*. To investigate changes in bacterial richness and homogeneity in mouse feces, we calculated the Shannon and evenness indices (the alpha-diversity indices). The Shannon (Figure 4b) and evenness indices (Figure 4c) in mice fed *Faecalibacterium* alone (F group) were significantly higher than those in mice fed *Bifidobacterium* alone (B group) (*p* < 0.05). However, the alpha-diversity between the F group and the FB group was not significantly different. We used the Bray-Curtis distance analysis to calculate the beta-diversity of samples in order to determine microbial phylogenetic similarity among the groups (Figure 4d). The structure of the microbial community in the control (*n*) group was similar to that in the F group, and the composition of the microbial community in the mice FB group) was similar to that in the B group.

Taxonomic analysis of the microbial composition at the genus level revealed different microbial composition in the four groups (Figure 4e). We also used the LEfSe analysis to identify bacteria specific for the treatment groups (Figure 4f). In the analysis, an LDA score greater than 2.0 reflects species with a significantly different abundance between the groups. *Faecalibacterium* was highly abundant in the FB microbiome, while Actinobacteria and Bifidobacteriales were enriched in the B group. The microbial communities in the *n* and FB groups were strikingly different. Finally, we determined the abundances of *Faecalibacterium* and *Bifidobacterium* genera in the feces by RT-qPCR (Figure 4g,h). *Faecalibacterium* was significantly more abundant in the FB group feces than in the F group feces (*p* < 0.01) (Figure 4g). Further, the feces from the FB and B groups contained significantly more bifidobacteria than those from the *n* and F groups (*p* < 0.01) (Figure 4h). However, bifidobacterial abundances in the FB and B group feces were not significantly different. 

### 3.3. Effect of Bacterial Culture Supernatant on Proinflammatory Cytokine Production by HT-29 Cells and RAW 264.7 Cells, and in the Colon in the DSS-Induced Colitis Mouse Model

**Figure 5:**
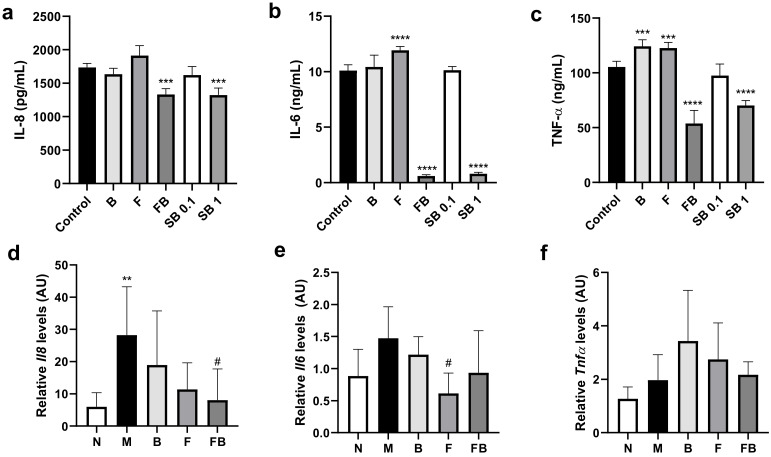
Immunomodulatory effects of bacterial culture supernatants *in vitro* (**a**–**c**) and *in vivo* (**d**–**f**). (**a**–**c**) *In vitro* effect of the culture supernatants: effect on the secretion of IL-8 by LPS-stimulated HT-29 cells (**a**), and on the secretion of IL-6 (**b**) and TNF-α (**c**) by LPS-stimulated RAW 264.7 macrophages. In **a**–**c**, the cells were exposed to sodium butyrate as the positive control. The cells were stimulated with 100 ng/mL LPS prior to the experiment, and cytokine levels were determined by using commercial enzyme-linked immunosorbent assay kits. (**d**–**f**) *In vivo* effect of the culture supernatants: relative mRNA levels determined by RT-qPCR for the genes of proinflammatory cytokines, *Il8, Il6,* and *Tnf-α,* in the colon of DSS-induced colitis mice. All data are shown as the mean + standard deviation. (**a**–**c**) Control, 10% YCFOS medium; B, 10% supernatant of *B. catenulatum* culture; F, 10% supernatant of *F. prausnitzii* culture; FB, 10% supernatant of *F. prausnitzii* and *B. catenulatum* co-culture medium; SB 0.1, 0.1 mM sodium butyrate; SB1, 1 mM sodium butyrate. Significant differences compared with the control group, *** *p* < 0.001, **** *p* < 0.0001 (*n* = 3). (**d–f**) N, untreated mice; M, DSS-induced colitis mice administered with culture medium control; B, DSS-induced colitis mice administered with medium from *B. catenulatum* culture; F, DSS-induced colitis mice administered with medium from *F. prausnitzii* culture; FB, DSS-induced colitis mice administered with medium from *F. prausnitzii* and *B. catenulatum* co-culture. Significant differences compared with the N group, ** *p* < 0.01; compared with the M group, # *p* < 0.05 (*n* = 6).

*F. prausnitzii* has anti-inflammatory effects in vitro and in vivo [4,5,6,7]. We first investigated the anti-inflammatory effects of FB (co-culture) supernatant against LPS-induced inflammation in vitro. To do this, we assayed inflammatory cytokine levels in HT-29 cells and RAW 264.7 cells exposed to different culture supernatants. As shown in Figure 5a, HT-29 cells treated with FB supernatant produced significantly less IL-8 than the negative control group (*p* < 0.001). IL-8 release was significantly lower upon exposure to the FB supernatant than that after exposure to either F or B supernatant (from monocultures). In addition, the effect of FB supernatant was similar to that of 1 mM sodium butyrate. Further, exposure to FB supernatant significantly blocked the release of LPS-induced cytokines IL-6 and TNF-α by RAW 264.7 macrophages (*p* < 0.0001; Figure 5b,c). As above, the effect of the FB supernatant was similar to that of 1 mM sodium butyrate. 

We also determined the effect of bacterial supernatant on the production of proinflammatory cytokines in vivo. Specifically, we used RT-qPCR to investigate changes in the expression of genes encoding IL-8, IL-6, and TNF-α cytokines in the colon of DSS-treated mice fed different bacterial culture supernatants. DSS treatment increased the *Il8* (*p* < 0.01) and *Il6* mRNA levels (M vs. *n* group in Figure 5d,e). In contrast, feeding FB supernatant significantly reduced the *Il8* mRNA levels compared with the control group treated with DSS (M group) (*p* < 0.05) (Figure 5d). Feeding F supernatant significantly reduced the *Il6* mRNA levels in the colon compared with control M group (*p* < 0.05). Feeding M, B, F, and FB supernatants did not significantly affect T*nf-α* mRNA levels.

### 3.4. Effect of Co-Culture Bacterial Supernatant on DSS-Induced Colitis in Mouse

**Figure 6:**
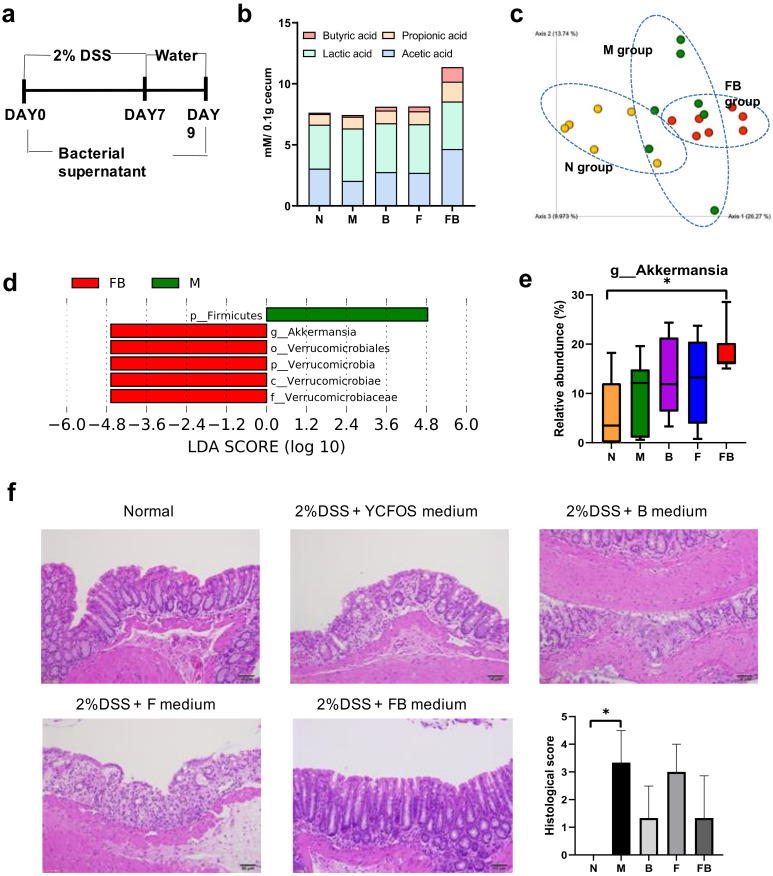
Effect of supernatant from bacterial co-culture on DSS-induced colitis in a mouse model. Colitis was induced in mice by feeding 2% (*w*/*v*) DSS for 7 d; the mice were concomitantly administered bacterial supernatant (**a**). The SCFA levels and microbiome were analyzed in mouse cecum samples (6 mice per group). The levels of butyrate, propionate, lactate, and acetate in the cecum are shown in (**b**). The beta-diversity based on the Bray-Curtis distance is shown in (**c**). Significantly different taxa in the FB (red) and M (green) groups were identified by LEfSe analysis (threshold >2.0), as shown in (**d**). Relative abundance of the genus *Akkermansia* is shown in (**e**). Histopathological changes in the colon are presented in (**f**). Representative H&E-stained colon sections are shown for each group, ×50 magnification, with the histological scores. The data are shown as the mean ± standard deviation (*n* = 6). Statistically significant differences vs. untreated mice (* *p* < 0.05). N, untreated mice; M, DSS-induced colitis mice administered culture medium control; FB, DSS-induced colitis mice administered with medium from *F. prausnitzii* and *B. catenulatum* co-culture; F, DSS-induced colitis mice administered with medium from *F. prausnitzii* culture; B, DSS-induced colitis mice administered with medium from *B. catenulatum* culture.

DSS-induced colitis is a commonly used model in IBD research. To evaluate the anti-inflammatory effect of the FB bacterial culture supernatant on DSS-induced colitis, we established DSS-induced colitis in mice (Figure 6a). The mice were fed ad libitum 2% (w/v) DSS in drinking water for 7 d, followed by normal tap water for 2 d; they were concomitantly fed the bacterial supernatant. At the end of the experiment, we determined the levels of four major SCFAs (acetate, propionate, lactate, and butyrate) in the cecum (Figure 6b). The cecum of mice fed the FB supernatant contained significantly more butyrate than the cecum of the *n* and M groups (*p* < 0.05). Further, the cecum of mice in the FB group contained significantly more acetate and propionate than the cecum in the M group. The Bray-Curtis distance analysis revealed distinct differences in the beta-diversity between the M, *n*, and FB groups (Figure 6c). We also used LEfSe to identify the dominant colonic microbiota in the M and FB groups. Pairwise comparison revealed an increased abundance of *Akkermensia* and Verrucomicrobiales in the FB group compared with the M group (Figure 6d). Further, feeding FB supernatant significantly increased the relative abundance of *Akkermansia* compared with the *n* group (*p* < 0.05; Figure 6e). Finally, as determined by H&E staining, feeding 2% DSS resulted in widely distributed intestinal epithelial damage, inflammatory cell infiltration, and edema in the colon (Figure 6f). In contrast, the intestinal epithelium in the FB group was relatively intact.

## 4. Discussion

Breakdown of microbial homeostasis can lead to IBD. *F. prausnitzii* is a good biomarker for a healthy gut, and its abundance is reduced in Crohn’s disease [14]. The bacterium induces the production of anti-inflammatory factors, such as IL-10, and produces butyrate. In the current study, we aimed to determine the factors that improve the growth and mammalian gut colonization of *F. prausnitzii*. We screened several bifidobacteria strains which have a nutritional cross-feeding relationship with *F. prausnitzii* to increase the viability of *F. prausnitzii* in the gut. We also investigated the anti-inflammatory effect of metabolites produced in *F. prausnitzii* co-culture with *Bifidobacterium* on intestinal inflammation in a DSS-induced colitis model.

IBD is a chronic inflammatory disease characterized by such clinical symptoms as weight loss, diarrhea, and rectal bleeding. In the current study, we induced IBD in mouse by administering DSS in drinking water. This IBD model is one of the best-characterized animal models that mimic human IBD symptoms. According to recent studies, live *F. prausnitzii* cells or the bacterial culture supernatant alleviate inflammation in DSS-induced colitis. However, because of the stringent growth conditions of *F. prausnitzii*, an extremely oxygen-sensitive bacterium, intestine delivery of viable cells is one of the current challenges precluding its use as a probiotic [8]. Indeed, we confirmed here that administration of *F. prausnitzii* culture supernatant from co-culture significantly increased the overall viability and colonization efficiency of *F. prausnitzii* and alleviated the colitis symptoms in mouse compared to culture supernatant from monoculture.

Bacteria in the large intestine compete for nutrients; however, metabolic byproducts secreted by one strain can be an essential energy source for another [37]. *F. prausnitzii* is reported to utilize the fermentation end-products of a few colonic bacteria including *Bacteroides thetaiotaomicron* and *E. coli* [26]. Bifidobacterial ability to utilize carbon sources is strain-specific, and these bacteria use various non-digestible carbohydrates, such as resistant starch, inulin, and FOS. Bifidobacteria that use a monosaccharides decomposition pathway as a main carbohydrate catabolism compete with *F. prausnitzii* for survival and growth [38]. However, *Bifidobacterium* strains capable of metabolizing non-digestible carbon sources, e.g., *B. catenulatum*, could support the carbohydrate availability of acetate-dependent butyrate producers such as *F. prausnitzii* by producing a high amount of acetate as an end-product in a nutrient-restricted environment [39]. In the present study, bifidobacteria, alone or in co-culture with *F. prausnitzii*, grew most rapidly during the initial 9 h of culture in a medium containing glucose, and during 9 to 24 h of incubation in a medium containing FOS. This suggests that the ability of bifidobacteria to degrade long-chain carbohydrates may affect the growth of other bacteria in co-culture. Based on the viable cell counts during growth on FOS, we showed that *B. catenulatum* KCTC 3221 grew similarly well in co-culture and monoculture. *F. prausnitzii* did not grow well during the first 9 h in co-culture with *B. catenulatum* KCTC 3221 but showed a 29-fold growth increase during the ensuing 9–24 h. It is feasible that the co-culture promoted the growth of *F. prausnitzii* by lowering the medium pH (to between 5.8 and 6.5) and supplying glycolysis metabolites, in addition to supplying acetate, as a result of carbon source degradation by *Bifidobacterium*. Improved butyrate production by *F. prausnitzii* in co-culture with *B. adolescentis* in the FOS-containing medium was reported previously [25]. In that study, bacterial growth was only determined by OD measurements, and it was hard to assess the growth-promoting ability of the strain. In order to improve the data interpretation, we followed the growth of each bacterium in co-culture by using viable cell counts as well. Further, we showed here that among the bifidobacterial strains tested, *B. catenulatum* KCTC 3221 most notably enhanced growth and butyrate production by *F. prausnitzii*. *B. catenulatum* is the most frequently detected species in adults and is present in 38% of individuals [40] and effective against acute liver damage [41,42]. Besides anti-inflammatory effects, butyrate has benefits to regulate pathological progress in metabolic diseases, including non-alcoholic fatty liver diseases and liver fibrosis. Further research investigating the effects of co-cultured *B. catenulatum* and *F. prausnitzii* on other types of inflammatory and metabolic diseases is required to evaluate the applicability as a potential candidate.

The above effects were strain-specific rather than characteristic for the entire *Bifidobacterium* genus or certain species. We here compared the bacterial growth rate (OD_650_) and SCFA levels in the cultures of four *B. animalis* strains. *B. animalis* subsp. *lactis* Ad011 grew rapidly during the first 9 h and produced copious amounts of lactate in monoculture and co-culture in most media tested. In a medium containing glucose, while *B. animalis* ATCC 25527 grew better than *B. animalis* subsp. *lactis* RD68 alone, the growth of both strains was similar in co-culture. However, the SCFA levels were different in co-cultures of these strains: *B. animalis* ATCC 25527 produced similar amounts of acetate and butyrate after 9 and 24 h, but *B. animalis* subsp. *lactis* RD68 produced less acetate and more butyrate after 24 h than after 9 h. This suggests that while moderate growth in co-culture on glucose of two different bacterial species has a synergistic effect on their growth, excessive growth of one bacterium can interfere with the growth of the other bacterium. In the FOS-containing medium, the co-cultures of *B. animalis* ATCC 25527 and *B. animalis* subsp. *lactis* RD68 with *F. prausnitzii* grew more rapidly in the period between 9 and 24 h than during the initial 9 h. On the other hand, the co-culture with *B. animalis* subsp. *lactis* SH5 showed similar growth in the two time periods. The co-culture with *B. animalis* subsp. *lactis* ad011 grew during the initial 9 h and then stopped. This emphasizes the notion that different strains of the same species show different growth characteristics on the same sugar source, not only in the monoculture, but also in co-culture with *F. prausnitzii*.

It is well-established that *F. prausnitzii* consumes acetate produced by Bifidobacteria and produces butyrate [24,25]. In the current study, we confirmed a decrease in acetate levels and an increase in butyrate levels after 24 h of culture, compared with those after 9 h. *F. prausnitzii* requires a carbohydrate energy source for growth and butyrate formation, and bifidobacteria do not produce butyrate. The observed changes in acetate and butyrate levels in co-cultures in the YCFOS medium not supplemented with SCFA suggest that the growth of *F. prausnitzii* was supported by acetate supplied by *Bifidobacterium*, while the two bacteria competed for FOS present in the culture medium. In contrast, during co-culture in the glucose- and starch-containing media, the acetate levels in the medium were higher after 24 h than those after 9 h. Carbon source utilization allowed for a rapid *Bifidobacterium* growth, with an increased production of acetate and the slowing of growth of *F. prausnitzii*, without a notable increase in butyrate levels. Hence, the interaction between the two bacteria depends on the type of carbon source in co-culture. Others have similarly reported increased butyrate levels and reduced acetate levels in *F. prausnitzii* co-culture with *Bifidobacterium* in FOS- or inulin-containing media [25,39,43]. FOS is a major source of carbon that can be used as an energy source by bacteria in the large intestine. It is not degraded in the upper digestive system, prior to passage to the large intestine [21]. In the current study, we confirmed that the bacteria grew better in a medium containing FOS than in a medium containing inulin, a longer-chain compound than FOS. *F. prausnitzii* and *B. catenulatum* produced the most butyrate in the FOS-containing medium.

To evaluate the intestinal delivery of *F. prausnitzii*, we orally administered the bacteria to healthy mice. In another study, live cultures of *F. prausnitzii* were fed to newborn calves to observe the effects on animal health and fecal microorganisms [42]. The group fed *F. prausnitzii* showed reduced rates of diarrhea and mortality. Further, fecal microbial analysis revealed that the relative abundance of *F. prausnitzii* and Firmicutes in feces increased 3 to 5 weeks since the start of bacterial feeding [44]. In the current study, based on the RT-qPCR analysis and fecal microbiome analysis, we showed that *Faecalibacterium* was significantly more abundant in the FB and F mice than in untreated mice. *Bifidobacterium* abundance was significantly higher in the FB and B groups than in untreated mice, while the relative abundance of *Faecalibacterium* was higher in the FB mice than in the F mice. *Faecalibacterium* does not easily colonize the intestine. However, ingestion of *Faecalibacterium* together with *Bifidobacterium* appeared to improve its intestinal colonization. As evidenced by the in vitro experiments described above, *Bifidobacterium* influences the growth of *Faecalibacterium*, which may explain the in vivo effects.

In the current study, we also evaluated the anti-inflammatory effect of FB bacterial supernatant in the colon of DSS-induced colitic mouse and on LPS-stimulated HT-29 cells and RAW 264.7 macrophages. The colonic *Il8* mRNA levels in the FB group were significantly lower than those in the DSS-induced control group. This was similar to the trend observed in the experiments with LPS-induced HT-29 colon cells. Further, FB culture supernatant inhibited TNF-α and IL-6 secretion by LPS-stimulated RAW 264.7 macrophages. LPS activates the innate immune system and induces the release of cytokines (TNF-α and IL-6) and chemokines (IL-8) [45]. According to previous studies, *F. prausnitzii* monoculture supernatant reduces NF-κB activation and IL-8 secretion in vitro [7,8,9,46]. However, the effect of supernatants of *F. prausnitzii* co-culture with *Bifidobacterium* on IL-8 levels has not been evaluated prior to the current study. The FB supernatant used for cell treatment in this study contained approximately 0.8–1.2 mM butyrate. Accordingly, the extent of cytokine level reduction elicited by the FB supernatant was similar to the effect of 1 mM sodium butyrate. Sodium butyrate inhibits the phosphorylation of AKT and the NF-κB P65 signaling pathway, with anti-inflammatory activity in mice treated with 2,4,6-trinitrobenzene sulfonic acid [15,47].

The effect of the *Faecalibacterium* supernatant on DSS-induced colitis in mice has been reported previously [48,49]. However, the ability of the supernatant to relieve inflammation in animals is controversial [11]. In the current study, the amount of butyrate in the cecum was significantly higher in the FB group than in other groups. This could be related to the amount of butyrate in the FB supernatant. The mechanism underpinning the effect of the FB supernatant could be explained by the reported beneficial effect of sodium butyrate on the abundance of *Akkermansia muciniphila*. Butyrate consumption decreases the pH in the colon, which stimulates the abundance of some bacteria, e.g., *Eubacterium cylindroides*. These bacteria promote mucin secretion within the mucus layer, which increases the abundance of *A. muciniphila* [50,51]. *Akkermensia* degrades the mucus, leading to a release of oligosaccharides and the production of propionate and acetate [52,53,54]. Analysis of the cecal microflora in the current study suggests that the relative abundance of *Akkermansia* in the FB group was significantly higher than that in the control group. *A. muciniphila* regulates the expression of genes involved in the host immunity, metabolism, and gut barrier functions. Both *A. muciniphila* and *F. prausnitzii* are present in the intestinal mucosa and are considered members of healthy gut microflora. The abundance of both species is reduced in several intestinal disorders (e.g., IBD). Therefore, the co-treatment of both live *F. prausnitzii* and *B. catenulatum* bacteria and supernatants can modulate the gut microbial community and alleviate the pathological progress of related diseases.

Although we here demonstrated the effectiveness of bifidobacteria in improving the intestinal delivery of *Faecalibacterium*, the study has some limitations. We showed that the growth of *F. prausnitzii* and bifidobacteria depends on the type of dietary fiber in vitro. Ingestion of FOS, a prebiotic, could further improve the delivery of *Faecalibacterium* to the intestine when the two bacteria are ingested together in vivo. However, because the composition of the large intestine of the mouse and human is different, further experiments are needed to determine whether colonization of *F. prausnitzii* in human would be similarly improved.

Taken together, we have shown here that the administration of *F. prausnitzii* together with bifidobacteria improves the intestinal delivery of the former. In addition, we showed that the co-culture of *F. prausnitzii* with bifidobacteria results in an increased production of butyrate, an immunomodulatory compound. Further, the supernatant from *Faecalibacterium* co-culture with bifidobacteria inhibited the production of proinflammatory cytokines in vitro and in vivo. Changes in the intestinal microflora upon supernatant administration were confirmed by 16S rRNA metagenomic analysis. *Bifiodbacterium* genus includes several probiotic strains, while *F. prausnitzii* is a good next-generation probiotic candidate. Administration of *F. prausnitzii* together with bifidobacteria shows great potential not only for maintaining intestinal homeostasis, but also as a treatment for IBD.

## Supplementary Materials

The following are available online at https://www.mdpi.com/2076-2607/8/5/788/s1: Figure S1. Growth of *F. prausnitzii* ATCC 27768 with different *Bifidobacterium animalis* strains in liquid culture in the presence of various carbohydrates. Figure S2. Production of SCFA by bacteria in monoculture and co-culture. Concentrations of SCFA (acetate, lactate, formate, and butyrate) were determined 9 and 24 h after inoculation. Figure S3. Changes in the viable cell number and pH of cultures of *F. prausnitzii* and *B. animalis* subsp. lactis RD68 in YCFOS medium.
