# Supplementary material for: Co-Culture with Bifidobacterium catenulatum Improves the Growth, Gut Colonization, and Butyrate Production of Faecalibacterium prausnitzii: In Vitro and In Vivo Studies
Source: Microorganisms. 2020 May 25;8(5):788. doi: 10.3390/microorganisms8050788 (PMC7285360; doi:10.3390/microorganisms8050788)
Supplement: Supplementary file 1 [file microorganisms-08-00788-s001.pdf]

Supplementary Materials: Figure S1. Growth of *F. prausnitzii* ATCC 27768 with different *Bifidobact erium animalis* strains in liquid culture in the presence of various carbohydrates. Figure S2. Pro duction of SCFA by bacteria in monoculture and co-culture. Concentrations of SCFA (acetate, l actate, formate, and butyrate) were determined 9 and 24 h after inoculation. Figure S3. Chang es in the viable cell number and pH of cultures of *F. prausnitzii* and *B. animalis* subsp. lactis RD68 in YCFOS medium.

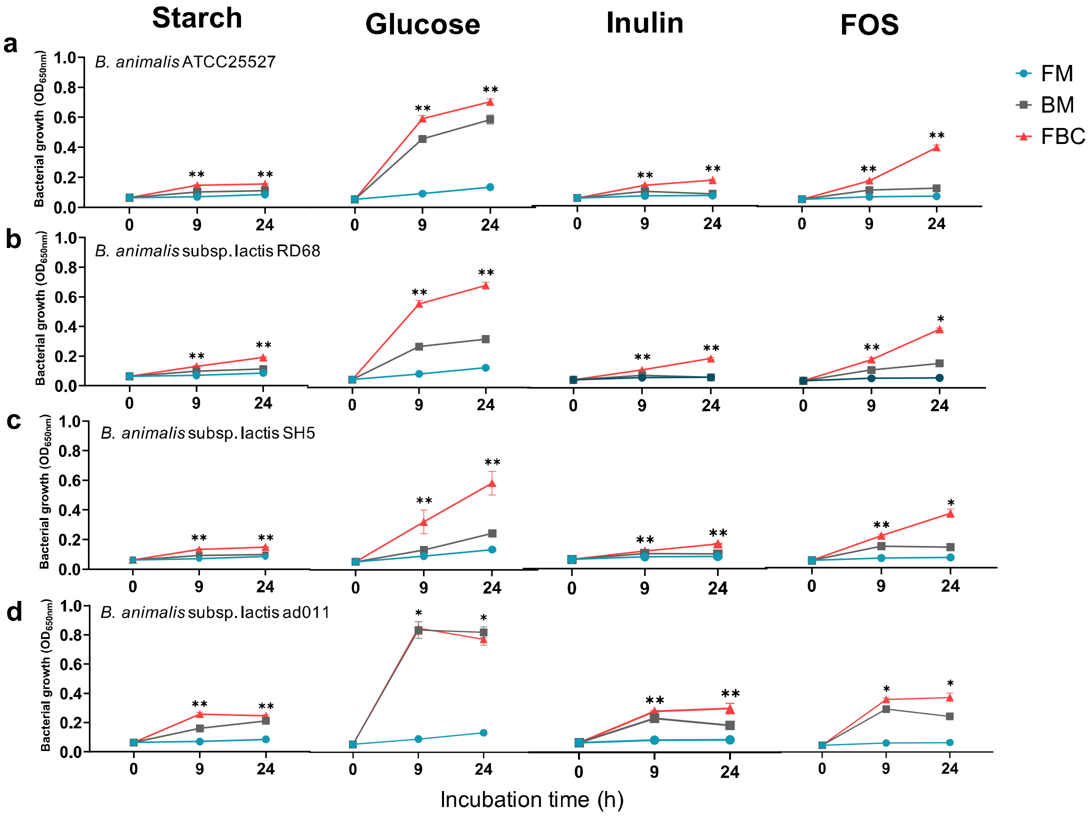

Figure S1. Growth of *F. prausnitzii* ATCC 27768 with different *Bifidobacterium animalis* strains in liquid culture in the presence of various carbohydrates. Culture OD_{650} was determined at various time points after inoculation. *F. prausnitzii* growth in monoculture and co-culture with *B. animalis* ATCC 25527 (**a**), *B. animalis* subsp. lactis RD68 (**b**), *B. animalis* subsp. lactis SH5 (**c**), and *B. animalis* subsp. lactis ad011 (**d**) is shown. All growth experiments were performed in the YCFA medium without SCFA supplemented with starch, glucose, inulin, or FOS, for 24 h. The data are presented as the mean ± standard deviation (n = 4). Statistically significant differences vs. FM group are denoted (**p* < 0.05, ***p* < 0.01). FM, monoculture of *F. prausnitzii*; BM, monoculture of *Bifidobacterium*; FBC, co-culture of *F. prausnitzii* and *Bifidobacterium*.

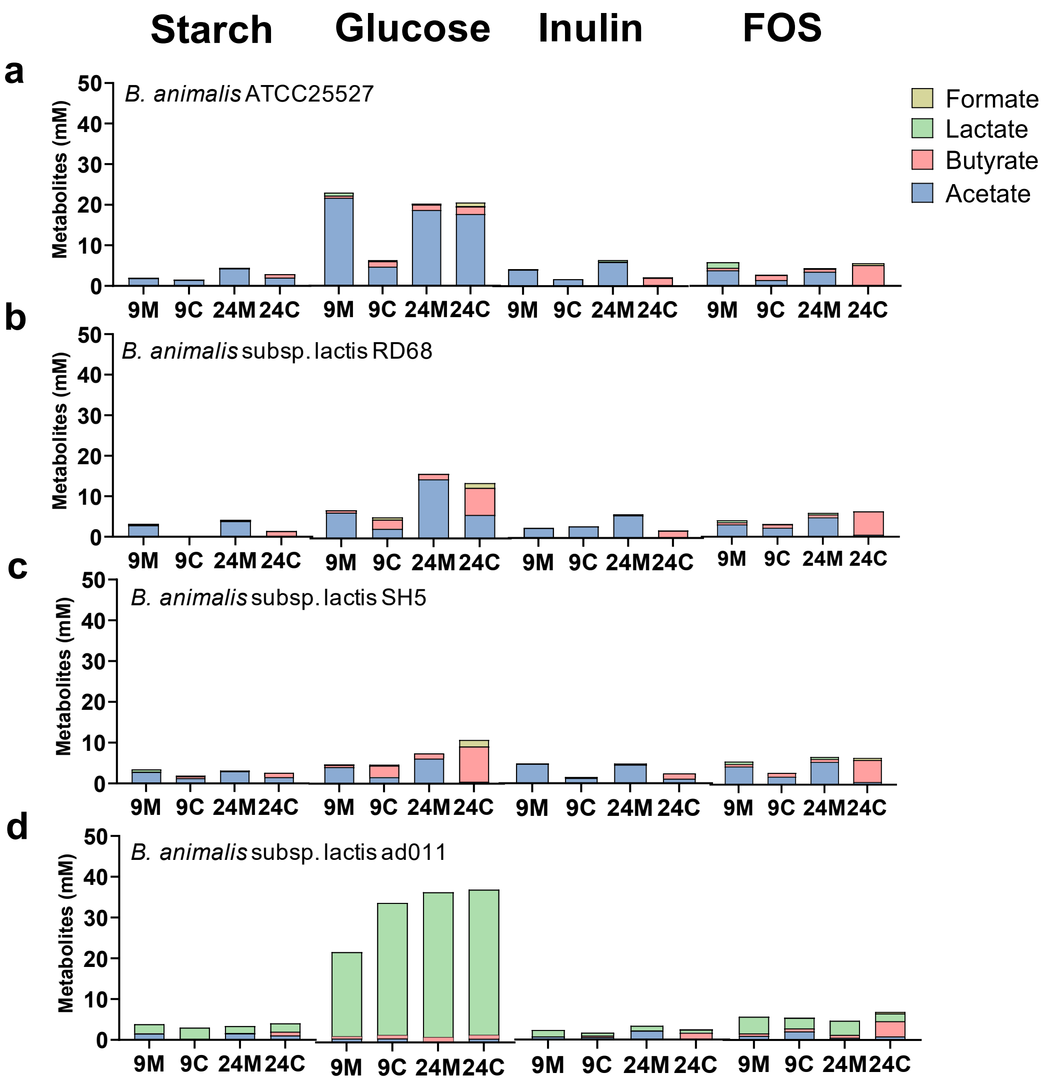

Figure S2. Production of SCFA by bacteria in monoculture and co-culture. Concentrations of SCFA (acetate, lactate, formate, and butyrate) were determined 9 and 24 h after inoculation. Data for *F. prausnitzii* in monoculture and in co-culture with *B. animalis* ATCC 25527 (**a**), *B. animalis* subsp. lactis RD68 (**b**), *B. animalis* subsp. lactis SH5 (**c**), and *B. animalis* subsp. lactis ad011 (**d**) are shown. All experiments were performed in YCFA medium without SCFA supplemented with starch, glucose, inulin, or FOS. The data are presented as the mean (n = 3). 9M, metabolites from *F. prausnitzii* and *Bifidobacterium* monocultures after 9 h; 9C, metabolites in *F. prausnitzii* and *Bifidobacterium* co-culture after 9 h; 24M, metabolites from *F. prausnitzii* and *Bifidobacterium* monocultures after 24 h; 24C, metabolites in *F. prausnitzii* and *Bifidobacterium* co-culture after 24 h.

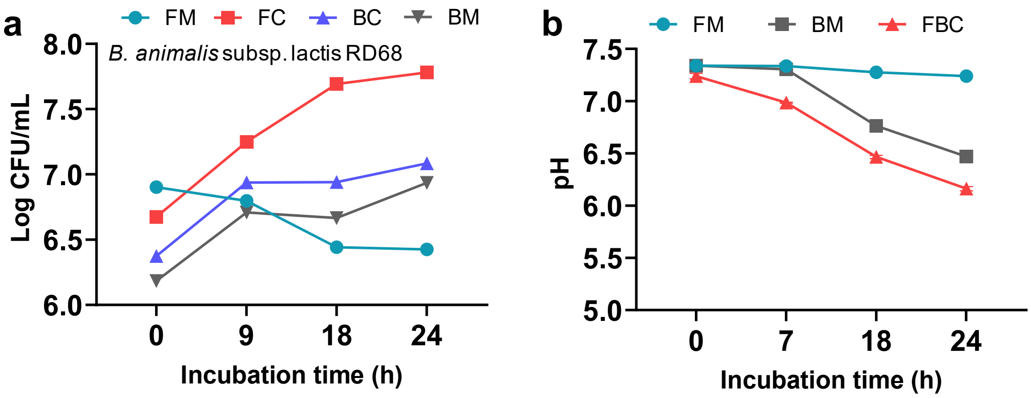

Figure 3. Changes in the viable cell number and pH of cultures of *F. prausnitzii* and *B. animalis* subsp. lactis RD68 in YCFOS medium. Data for *F. prausnitzii* in monoculture or co-culture with *B. animalis* subsp. lactis RD68 (**a**,**b**) in YC medium supplemented with FOS are shown. Samples were analyzed at the indicated time intervals. Cell numbers were determined by viable counts, as CFU/mL, after plating on LYBHI agar (**a**). The pH changes are plotted in (**b**). The data are presented as the mean ± standard deviation (n = 3). FM, *F. prausnitzii* in monoculture; BM, *Bifidobacterium* in monoculture; FC, *F. prausnitzii* in co-culture; *BC, Bifidobacterium* in co-culture; *FBC, co-culture of F. prausnitzii and Bifidobacterium.*
